# Spectral Efficiency Optimization of Uplink Millimeter Wave MIMO-NOMA Systems

**DOI:** 10.3390/s22176466

**Published:** 2022-08-27

**Authors:** Yinhao Zhang, Honggui Deng, Jun He, Zaoxing Zhu, Chengzuo Peng, Haoqi Xiao

**Affiliations:** School of Physics and Electronics, Central South University, Changsha 410083, China

**Keywords:** spectral efficiency, millimeter wave, MIMO, NOMA, user grouping, power allocation

## Abstract

In this paper, we considered uplink communication, focusing on the improvement of spectral efficiency (SE) for millimeter wave (mmWave) multiple-input multiple-output non-orthogonal multiple access (MIMO-NOMA) systems. Firstly, we proposed an adaptive cluster head selection algorithm. Then, a channel-aligned analog beamforming scheme was designed based on the selected cluster heads. After that, the user grouping algorithm was designed based on the user-equivalent channel correlation. Subsequently, the power allocation problem was transformed from a nonconvex problem to a convex one using the quadratic transformation (QT) method considering all relevant constraints. Finally, the optimal user power allocation and digital beamforming design was obtained by iteratively optimizing the power and digital beamforming. Simulation results show that our proposed scheme can achieve a higher SE than existing methods.

## 1. Introduction

Non-orthogonal multiple access (NOMA) is one of the core technologies of next-generation wireless communications, and combined with conventional millimeter wave (mmWave) multiple-input multiple-output (MIMO) it can significantly improve the spectral efficiency (SE) of the system [1,2]. NOMA can transmit signals for different users in the same time-frequency domain while differentiating in the power domain. By using superimposed coding at the transmitter and successive interference cancellation (SIC) at the receiver, it is possible to simultaneously serve users with different channel conditions, thus improving the SE of the system [3,4].

However, there are two serious technical obstacles to mmWave MIMO-NOMA communication systems. The first problem is that the number of users is much larger than the number of radio-frequency (RF) chains, leading to serious inter-user interference [5,6]. The other problem is the dramatic increase in the number of users in next-generation communication systems, which increases energy consumption and leads to the power allocation problem [7,8]. Therefore, inter-user interference and power allocation are two fundamental challenges that need to be addressed in next-generation communication systems.

The existing studies contribute to resolving the above problems by designing a user grouping algorithm and power allocation algorithm.

Ref. [9] used channel gain difference as an indicator for user grouping and derived a closed-form solution for optimal power allocation for arbitrary clusters. However, mmWave systems are characterized by high spatial directionality, and a user grouping scheme considering only channel gain cannot yield an optimal user grouping strategy. Therefore, in [10,11], user grouping was performed based on channel correlation and gain differences, and an iterative power allocation scheme was used to maximize the system performance under different constraints. However, refs. [10,11] are 2-user grouping schemes. To break the limitation of the number of users in the same group, refs. [12,13,14,15] extended the number of user groupings from 2-user to K-user. Ref. [12] studied the downlink mmWave MIMO-NOMA system and proposed a cluster head selection algorithm for user groupings, considering synchronous radio information and power transfer (SWIPT). Based on  [12,13] proposed a K-means grouping algorithm by splitting the power allocation problem into intra-group and inter-group power allocation to solve it. However, the traditional K-means algorithm is unstable because of the random selection of initial cluster head users. Ref. [14] selected the initial cluster head users based on channel gain and used an improved K-means algorithm to reduce the impact of initial cluster head selection on system performance. In [15], an inter-cluster and intra-cluster power allocation scheme was used to further improve the system SE based on the improved K-means algorithm combined with the simulated beamforming design. Ref. [16] considered the power allocation problem of a downlink NOMA system, and optimized the user scheduling, time slot allocation and power control to maximize the α-fairness of the system. In the presence of a successive interference cancellation (SIC) error over Rayleigh fading channels in  [17], the authors investigated the downlink NOMA system performance analysis and derived the exact closed-form expressions of the bit error rate (BER) for the considered downlink NOMA system at near-user and far-user. Ref. [18] discussed a downlink MIMO-NOMA cooperative two-user system. In addition, a SWIPT assisted MIMO-NOMA cooperative transmission strategy model incorporating self interference and beamforming was proposed to improve the data rate fairness and outage performance of the cell-edge user. Refs. [12,13,14,15,16,17,18] studied the system performance design of downlink communication.

For the study of the uplink mmWave MIMO-NOMA system, ref. [19] proposed user grouping based on the location of the user detected by the base station (BS), and an iterative power allocation and hybrid beamforming update scheme. Ref. [20] considered imperfect channel state information (CSI) and proposed two different user grouping schemes, i.e, non-scalable far and near grouping and scalable neighbor grouping. Refs. [19,20] minimized the user power by user grouping and power allocation scheme design. Ref. [21] proposed a novelinitial agglomerative nesting (AGNES) user grouping algorithm combined with an analog beamforming design scheme, considering the beam overlap problem to maximize SE through iterative digital beamforming and power allocation.

In this paper, we studied the maximization of the SE for an uplink mmWave MIMO-NOMA system. We designed a novel joint optimization framework, which includes user grouping, hybrid beamforming and power allocation. The main contributions of this paper are summarized as follows

- Aiming at solving the problem of inter-user interference, we proposed an adaptive cluster head selection algorithm based on channel gain and channel correlation, which suppresses inter-user interference by selecting cluster head users.
- Aiming at the problem of inter-beam interference, we proposed analog beamforming based on user channel alignment to solve the problem of inter-beam interference and further improve the SE of the system.
- For the user power allocation problem, the quadratic transformation (QT) method was used to transform a non-convex power allocation problem into a solvable convex problem. Then an iterative approach was designed to obtain optimal power allocation and digital beamforming.
- From the perspective of SE, the proposed joint optimization framework was evaluated by performing simulations. The simulation results demonstrate that our proposed algorithms can significantly enhance SE compared to existing methods by a variety of simulation parameters. Under the parameter setting of this article, the algorithm of this paper outperforms traditional algorithm by about 54.6%.

The remainder of this paper is organized as follows. Section 2 describes the system model of uplink mmWave MIMO-NOMA and SE maximization problem for this system. Section 3 details the solution strategy, including a user grouping process based on cluster head selection, HBF and an optimized power allocation algorithm. In Section 4, the simulation results are provided to demonstrate the performance. Finally, Section 5 concludes the paper and provides an outlook for the future.

The notations used in this paper are listed as follows. $\mathrm{CN}(0,\sigma^{2}I)$ denotes the circularly symmetric complex Gaussian (CSCG) distribution. Superscripts ${\left(\cdot \right)}^{-1}$,${\left(\cdot \right)}^{T}$ and ${\left(\cdot \right)}^{H}$ denote the inversion, the transpose and the conjugate transpose of a matrix or vector, respectively. $C^{a\times b}$ denotes the space of a×b complex-valued matrices. ${∥\cdot ∥}_{2}$ denotes the Frobenius norm of a matrix or vector. |·| denotes the absolute value of a scalar or the cardinality.

## 2. System Model and Problem Formulation

### 2.1. System Model

**Figure 1:**
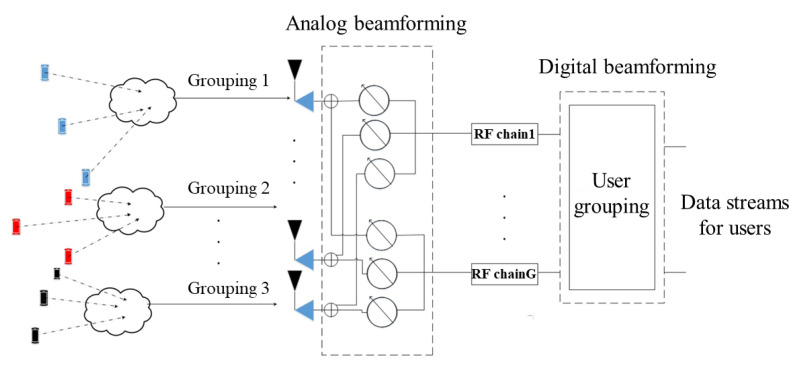
System model of uplink mmWave-MIMO-NOMA communications.

We considered an uplink MIMO-NOMA system with K single-antenna users communicating with the BS, where a fully connected hybrid precoding structure with $N_{BS}$ number of antennas and $N_{RF}$ number of RF chains was used at the BS to receive the signals transmitted by the users (the user set was defined as $U=\left\{U_{1},U_{2},\ldots ,U_{K}\right\}$). The system model is shown in Figure 1. Users transmit the data stream through the antenna, which is processed by analog precoding $F_{RF}$ and digital precoding $F_{BB}$ and then arrived at the BS.

To obtain higher multiplexing gain, we assumed the number of RF chains $N_{RF}$ to be equal to the number of data streams *G*.  K users were divided into *G* user groups, and each group corresponds to a separate data stream. After user grouping, the *g*-th user group was denoted as $S_{g}=\left\{U_{g,1},U_{g,2},\ldots ,U_{g,\left|S_{g}\right|}\right\}$, and  $\left|S_{g}\right|>1$ with $\sum_{g=1}^{G}\left|S_{g}\right|=K$. After the next power allocation process for each user in each group (see subsequent sections for the specific power allocation scheme), each user transmits signals with their assigned power, which are finally transmitted to the BS. The BS will receive the signals which can be presented as

(1) $$R=\sum_{g=1}^{G}\sum_{u=1}^{\left|S_{g}\right|}\sqrt{P_{g,u}}\;h_{g,u}x_{g,u}+n,$$

where $U_{g,u}$ is the *u*-th user of group *g*, $h_{g,u}\in C^{N_{BS}\times 1}$ is the channel matrix of $U_{g,u}$, $P_{g,u}$ is the user power assigned to $U_{g,u}$, $x_{g,u}$ is the signal sent by $U_{g,u}$, and n is the noise obeying the complex Gaussian distribution $\left(n_{g,u}∼CN\left(0,\sigma^{2}I\right)\right)$. Thereafter, these signals are also subjected to hybrid beamforming processing and finally accepted by the BS. The expression of the accepted signal after processing by the BS is as follows

(2) $$Y=F_{BB}^{H}\;F_{RF}^{H}\sum_{g=1}^{G}\sum_{u=1}^{\left|s_{g}\right|}\sqrt{P_{g,u}}\;h_{g,u}x_{g,u}+F_{BB}^{H}\;F_{RF}^{H}n,$$

where $F_{\mathrm{RF}}\in C^{N_{\mathrm{BS}}\times G}$ represents the analog precoding matrix, in which each element of the matrix is constrained by the unit modulus, i.e., ${\left|F_{RF}^{\left(i,j\right)}\right|}^{2}=\frac{1}{N_{BS}}$. $F_{BB}=\left[f_{1}^{BB},\ldots ,f_{G}^{BB}\right]\in C^{N_{BS}\times G}$ represents the digital precoding matrix.

The channel model of mmWave has rich geometric features because it propagates basically along a straight line, has poor bypass capability and fewer scattering paths in its channel. Based on this property without loss of generality, we modeled it using the Saleh–Valenzuela model, assuming a uniform line array (ULA) with half-wavelength antenna spacing  [22]. In this model, the channel matrix for the *u*-th user in the *g*-th group is as follows

(3) $$h_{g,u}=\sqrt{\frac{N_{BS}}{\;L}}\left(\alpha_{g,u,0}\;h_{g,u,0}+\sum_{l=1}^{L}\alpha_{g,j,l}\;h_{g,u,l}\right),$$

in the above equation, *L* is the number of the propagation paths of $U_{g,u}$, $h_{g,u,0}$ and $h_{g,u,1}$ are the line-of-sight (LoS) channel matrix and the *l*-th non-line-of-sight (NLoS) channel matrix with $\alpha_{g,u,0}$ and $\alpha_{g,u,1}$ denoting the corresponding complex gains. The specific expression of $h_{g,u,l}$ is as follows

(4) $$h_{g,u,l}=a_{BS}\left(N_{BS},\theta_{g,u,l}\right),$$

(5) $$a_{BS}\left(N_{BS},\theta_{g,u,l}\right)=\frac{1}{\sqrt{N_{BS}}}{\left[1,e^{j\frac{2\pi d}{\lambda }\cos\left(\theta_{g,u,l}\right)},\ldots ,e^{j\frac{(N-1)2\pi d}{\lambda }\cos\left(\theta_{g,u,l}\right)}\right]}^{T},$$

in the above equation, $a_{BS}\left(N_{BS},\theta_{g,u,l}\right)\in C^{1\times N_{BS}}$ is the normalized receiver array response vectors, where $\theta_{g,u,l}\in \left[-\frac{\pi }{2},\frac{\pi }{2}\right]$ is the angle-of-arrival (AOA) of $U_{g,u}$ at the *l*-th path. *d* is the antenna spacing and λ is the wavelength. For a half-wavelength antenna spacing array, we have $d=\frac{\lambda }{2}$.

### 2.2. Problem Formulation

In uplink mmWave MIMO-NOMA systems, the decoding order of users at the BS can impact the system performance. Therefore, it is necessary to sort the user-effective channel gain firstly and determine the decoding order  [23]. We assumed that the user-effective channel gain ordering was as follows

(6) $${∥F_{RF}^{H}\;h_{g,1}∥}_{2}^{2}\geq {∥F_{RF}^{H}\;h_{g,2}∥}_{2}^{2}\geq \cdots \geq {∥F_{RF}^{H}\;h_{g,\left|s_{g}\right|}∥}_{2}^{2},$$

When decoding is performed, the strongest signals are decoded firstly, while the remaining weaker signals that are not decoded are considered as interference. We delete the decoded signals from them and continue to decode the remaining signals. The above process is repeated until all users are finished decoding. Therefore, the signal to interference plus noise power ratio (SINR) of $U_{g,u}$ can be written as

(7) $${\mathrm{SINR}}_{g,u}=\frac{{∥{\left(f_{g}^{BB}\right)}^{H}\;F_{RF}^{H}\;h_{g,u}∥}_{2}^{2}P_{g,u}}{I_{g,u}^{\mathrm{intra}\;}+I_{g,u}^{\mathrm{inter}\;}+{∥{\left(f_{g}^{BB}\right)}^{H}\;F_{RF}^{H}∥}_{2}^{2}\sigma^{2}},$$

(8) $$I_{g,u}^{\mathrm{intra}\;}=\sum_{v=u+1}^{\left|S_{g}\right|}{∥{\left(f_{g}^{BB}\right)}^{H}\;F_{RF}^{H}\;h_{g,v}∥}_{2}^{2}P_{g,v},$$

(9) $$I_{g,u}^{\mathrm{inter}\;}=\sum_{q\neq g}^{G}\sum_{v=1}^{\left|S_{q}\right|}{∥{\left(f_{g}^{BB}\right)}^{H}\;F_{RF}^{H}\;h_{q,v}∥}_{2}^{2}P_{q,v},$$

where $I_{g,u}^{\mathrm{intra}\;}$ and $I_{g,u}^{\mathrm{inter}\;}$ are the intra-user group interference and inter-user group interference, respectively. After obtaining the expression for SINR of $U_{g,u}$, the achievable rate of $U_{g,u}$ can be expressed as

(10) $$R_{g,u}=\log_{2}\left(1+SINR_{g,u}\right),$$

This paper focused on the SE maximization of the system, and the sum data rate of the system can be expressed as

(11) $$SE=\sum_{g=1}^{G}\sum_{u=1}^{\left|S_{g}\right|}R_{g,u},$$

thus, the problem of maximizing the SE of the system can be expressed as

(12) $$\begin{array}{cc} \; & \max_{\left\{P_{g,u}\right\}}SE\\ \; & \;s.t.C1\;:\;P_{g,u}\leq P_{g,u}^{\max},\\ \; & \;\;\;\;\forall g=1,2,\ldots ,G,u=1,2,\ldots ,\left|S_{g}\right|,\\ \; & \;\;\;C2:R_{g,u}\geq R_{\min},\\ \; & \;\;\;\;\forall g=1,2,\ldots ,G,u=1,2,\ldots ,\left|S_{g}\right|,\;\\ \; & \;\;\;C3:{∥{\left(f_{g}^{BB}\right)}^{H}\;F_{RF}^{H}\;h_{g,u}∥}_{2}^{2}P_{g,u}-\\ \; & \;\;\;\;\sum_{r=u+1}^{\left|s_{g}\right|}{∥{\left(f_{g}^{BB}\right)}^{H}\;F_{RF}^{H}\;h_{g,r}∥}_{2}^{2}P_{g,r}\geq P_{\mathrm{tol}\;},\\ \; & \;\;\;\;\forall g=1,2,\ldots ,G,u=1,2,\ldots ,\left|S_{g}\right|-1, \end{array}$$

where C1 represents the power maximum power constraint for each user, C2 is the minimum rate constraint that each user needs to achieve, and C3 is used to ensure that the user has sufficient clearance for the final power gain to ensure successful SIC decoding. $P_{\mathrm{tol}}$ is the minimum power difference required to distinguish the desired decoded signal from the remaining undecoded interfering codes in the group  [19]. For the above maximizing system SE, a solution incorporating user grouping, hybrid beamforming and user power allocation are proposed in the next section of this paper.

## 3. Solution Approach

**Figure 2:**
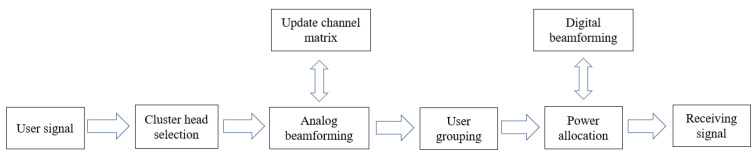
The framework of System model.

This section proposes a solution strategy for the problem described in (12). Firstly, an adaptive cluster head selection algorithm is proposed to design a channel-aligned analog beamforming based on the selected cluster heads and to update the channel matrix, followed by user grouping based on the user-equivalent channel correlation. Then, the zero-forcing (ZF) precoding is used to design the digital beamforming. Finally, the QT method is transformed from a non-convex power allocation problem to a convex problem. The optimal user power allocation and digital beamforming are obtained by iteratively optimizing the power and digital beamforming. (See Figure 2 for the general flow).

### 3.1. Adaptive Cluster Head Selection Algorithm

In the mmWave MIMO-NOMA system, the number of users K is more than the number of RF chains $N_{RF}$, which brings about the user interference problem and leads to the reduction of the SE of the system. In this subsection, an adaptive cluster head selection algorithm is designed for the interference problem of the user group.

The cluster head user is the representative user of each user group. For the cluster head user selection principle, the cluster head user is the user with the highest channel gain in each user group, and the channel correlation between different cluster head users is low. For the user channel correlation between high and low judgment, an adaptive threshold δ is introduced to measure the channel correlation of the cluster heads [12]. In the proposed adaptive cluster head selection algorithm, we firstly calculate the channel gain of all users to sort and select the user with the highest channel gain into the cluster head user set. After selecting the first cluster head user, the channel correlation between the first cluster head user and the remaining users is calculated. The users with less than a threshold δ are considered the candidate cluster head user set. The expression for calculating the user channel correlation is as follows

(13) $$\varphi \left(i,j\right)=\frac{\left|h_{i}\;h_{j}^{H}\right|}{\left|h_{i}\right|\left|h_{j}\right|},$$

where i represents the selected cluster head user and j represent the remaining users in the cluster head user group. $h_{i}$ and $h_{j}$ denote the channel matrix of user i and user j, respectively.

After obtaining the cluster head of the first beam, the the user with the highest channel gain from the set of candidate cluster heads needs to be selected as the cluster head for the second beam. After that, we calculate the channel correlation between the cluster head user of the second beam and the remaining users other than the cluster head user settings, and select the users with less than the threshold δ to update the candidate cluster head user set. Then the above process is repeated to select the cluster head of the third beam until there are no users in the candidate user set (Algorithm 1 provides the details of cluster head selection). To reasonably select candidate users, we set the threshold δ as an adaptive parameter and update the threshold by adding a small increment. Each threshold update is as follows

(14) $$\delta =\delta +\frac{\left(1-\delta \right)}{10},\delta \in \left(0,1\right),$$

**Algorithm 1** Adaptive cluster head selection method**Input:** The number of users *K*, and the number of beams *G*, channel veactors: $h_{i}$, $i\in \left[1,K\right]$, adaptive threshold: δ. **Output:** The cluster head set $∁={∁}_{1},{∁}_{2},\ldots ,{∁}_{G}$.  1: Calculate channel gains for each user and Sort in descending order, Note O;  2: Selecct the user O(1) with the highest channel gain to ∁;  3: $\rho =\frac{O}{∁}$;  4: p=ρ;  5: g=2;  6: **while **g<=G** do**  7:   **if** p==Φ **then**  8:     **while** p==Φ **do**  9:        Update adaptive threshold δ in accordance with (14) 10:        $\varphi \left(i,j\right)=\frac{\left|h_{i}h_{j}^{H}\right|}{\left|h_{i}\right|\left|h_{j}\right|},\;i\in \rho ,\forall j=p$; 11:        $p=\varphi \left(i,j\right)<\delta$; 12:     **end while** 13:   **end if** 14:   p={φ(i,j)<δ}i∈ρ,∀j=p; 15:   $∁=∁\cup p\left(1\right)$; 16:   $\rho =\frac{O}{∁};g=g+1$; 17: **end while**


### 3.2. Hybrid Precoding and User Grouping

After the cluster head user selection, the analog beamforming needs to be designed to reduce the inter-beam interference problem. The inter-beam interference reduction problem is the process of maximizing the antenna array by aligning the phases of the user channel matrix. Therefore, this paper proposed a phase-aligned analog beamforming design. Considering *B* bits quantized phase shifters, the non-zero elements in the analog precoding matrix $F_{\mathrm{RF}}=\left[f_{1}^{RF},\ldots ,f_{G}^{RF}\right]\in C^{N_{\mathrm{BS}}\times G}$ is in the codebook *D* as follows:

(15) $$D=\frac{1}{\sqrt{N_{BS}}}\left\{e^{j\frac{2\pi n}{2^{B}}}\right\},n=0,1,\ldots ,2^{B}-1,$$

where the phase of the codebook vector *D* is $\gamma =\left\{\frac{2\pi n}{2^{B}},n=0,1,\ldots ,2^{B-1}\right\}$. Next, the phase of the selected cluster head user in the previous subsection needs to be extracted. Then, the difference between the phase of the cluster head user and γ needs to be calculated, and the phase difference needs to be minimized to achieve the maximum gain of the antenna array. The specific expression is as follows

(16) $$\hat{n}=\underset{n\in \left\{0,1,\ldots ,2^{B}-1\right\}}{\mathrm{argmin}}|\;\mathrm{angle}\;\left(h_{g}\left(i\right)\right)-\frac{2\pi n}{2^{B}}|,$$

where $\;\mathrm{angle}\;\left(h_{g}\left(i\right)\right)$ represents the *i*-th phase element of the user channel matrix of the *g*-th cluster head selected in the previous section, $i=1,2,\ldots ,N_{\mathrm{BS}}$, g=1,2,…,G. According to the principle of minimizing the phase difference, we can update the simulated precoding vector elements one by one as follows

(17) $$f_{g}^{RF}\left(i\right)=\frac{1}{\sqrt{N_{t}}}e^{j\frac{2\pi \hat{n}}{2^{B}}},$$

After the analog beamforming design is completed, the equivalent channel for K users needs to be updated at this point, as demonstrated in the following equation:

(18) $${\bar{h}}_{k}^{H}=h_{k}^{H}F_{RF},$$

where k=1,…,K. The remaining users are then assigned to the appropriate cluster head user group based on the updated equivalent channel matrix. To improve the multiplexing gain of users, we performed the user grouping process based on the principle of inter-user channel correlation. Then, we calculated the channel correlation between the cluster head user group and the remaining users and assigned the users to the highly correlated user group. The specific channel correlation can be expressed as

(19) $$\hat{G}=\frac{\left|{\bar{h}}_{m}^{H}{\bar{h}}_{C_{\left(g\right)}}^{H}\right|}{∥{\bar{h}}_{m}∥∥{\bar{h}}_{C_{\left(g\right)}}∥}\left(g\in \left\{1,\ldots ,G\right\}\right),$$

where *m* represents the remaining users in the removed cluster head group, and user $C_{\left(g\right)}$ represents the *g*-th cluster head user among the selected cluster head group users. The details of the grouping scheme are provided in Algorithm 2. In Step 1–Step 2, we set the number of remaining users to be selected, which is denoted as *Reuser*. In Step 3–Step 5, the equivalent channel correlation between the cluster head user and the remaining users is calculated, and the user with the highest channel correlation with the cluster head user is placed in the most suitable cluster head group $\hat{g}$ in Step 6–Step 10, and the above algorithm is repeated until the remaining users are assigned.
**Algorithm 2** The algorithm of attributing users to clusters**Input:** The number of users *K*, and the number of beams *G*, channel matrix: $H=[h_{1},\ldots ,h_{k}]$, $k\in \left[1,K\right]$, The cluster head set $∁={∁}_{1},{∁}_{2},\ldots ,{∁}_{G}$, number of RF chains $N^{RF}$, number of BS antennas *N*, number of quantized phase shifters: B bits. **Output:** The user grouping scheme: $U=U_{1},U_{2},\ldots ,U_{G}$. **for **$l=1\to \left(Reuser\right)$** do**    {Reuser represents the number of users except for the cluster header user}    **for** m=1→G **do**      Calculate user-equivalent channel correlation by (18);    **end for**    $\hat{g}=\underset{g\in \{1,2,\cdots ,G\}}{\arg\max}$$\hat{G}$;    $use_{g}=U\left(\hat{g},:\right)$;    $use_{g}\left(use_{g}==0\right)=\left[\right]$;    $U\left(\hat{g},:\right)=U\left(\hat{g},:\right)\cup$Reuser(l); **end for**

After the analog beamforming design and user grouping process, the digital beamforming needs to be designed to eliminate the inter-beam interference and select the users with the strongest equivalent channel in each beam (we mention the equivalent channel in Equation (18)). This paper used a low-complexity ZF precoding scheme to reduce the inter-beam interference  [8]. Firstly, we initialized the power allocation by allocating the maximum power value $P_{max}$ to all users and then sorted and classified the users according to their channel and analog beam gain as follows: ${∥F_{RF}^{H}\;h_{g,1}∥}_{2}^{2}\geq {∥F_{RF}^{H}\;h_{g,2}∥}_{2}^{2}\geq \ldots \geq {∥F_{RF}^{H}\;h_{g,\left|s_{g}\right|}∥}_{2}^{2}$, g=1,…,G. Then, the user-equivalent channels were arranged in descending order as follows: $\dot{H}=\left[{\dot{h}}_{1},{\dot{h}}_{2},\ldots ,{\dot{h}}_{G}\right]$, where ${\dot{h}}_{g}=\sqrt{P_{g,1}}\;F_{RF}^{H}h_{g,1}$. According to the principle of maximizing user channel gain, we need to select the user with the strongest channel gain in all groups as the center of mass of the beam, and design the digital beam formation based on the strongest user. Finally, the inter-beam interference was eliminated by the ZF digital precoding method to obtain $F_{BB}$

(20) $$F_{BB}=\dot{H}{\left({\dot{H}}^{H}\dot{H}\right)}^{-1},$$

Since the digital precoding matrix is limited by unit power, each column of the digital precoding matrix should be normalized according to the following requirements

(21) $$f_{g}^{\mathrm{BB}}=\frac{f_{g}^{\mathrm{BB}}}{∥F_{\mathrm{RF}}f_{g}^{\mathrm{BB}}∥},g=1,\ldots ,G,$$

after obtaining the digital precoding matrix of our design by eliminating inter-beam interference through the ZF precoding scheme, we reordered the users according to the descending order of the equivalent channel gain as follows

(22) $${∥{\left(f_{g}^{BB}\right)}^{H}\;F_{RF}^{H}\;h_{g,1}∥}^{2}\geq {∥{\left(f_{g}^{BB}\right)}^{H}\;F_{RF}^{H}\;h_{g,2}∥}^{2}\geq \cdots \geq {∥{\left(f_{g}^{BB}\right)}^{H}\;F_{RF}^{H}\;h_{g,\left|s_{g}\right|}∥}^{2}.$$

### 3.3. Power Allocation

In this section, a power allocation scheme is proposed to distribute the power to each user in the beam in the most optimal way possible. To optimize the SE of the mmWave MIMO-NOMA system under the constraint of Quality of Service (QoS), we present the power optimization problem which is nonconvex. This paper used the QT method to transform nonconvex problems into solvable problems with low computational complexity [24].

Since the objective function in problem (12) is fractional in structure, the problem is a more difficult nonconvex problem to solve. Therefore, problem (12) is simplified. Firstly, we rewrite the constraint C2

(23) $$\begin{array}{c} C2:D_{g}\left(g,u\right)P_{g,u}-\left(2^{R_{\min}}-1\right)\\ \left(\sum_{U_{q,v}\in \Omega_{g,u}}d_{g}\left(q,v\right)P_{q,v}+\sigma^{2}\right)\geq 0, \end{array}$$

where $d_{g}\left(g,u\right)={∥{\left(f_{g}^{BB}\right)}^{H}F_{RF}^{H}h_{g,u}∥}^{2}$. $d_{g}\left(g,u\right)$ represents the equivalent channel gain of the *u*-th user in group *g* after beam selection and digital beamforming design. The $\Omega_{g,u}$ user set includes users in the same group whose channel gain is weaker than $U_{g,u}$ and all users in other groups. Referring to the substitution of the constraint in C2, the constraint in C3 can be written in the same way as follows

(24) $$C3:D_{g}\left(g,u\right)P_{g,u}-\sum_{r=u+1}^{\left|S_{g}\right|}d_{g}\left(g,r\right)P_{g,r}\geq P_{tol},$$

After changing the constraints C2 and C3, (12) can be transformed into (25) as follows

(25) $$\begin{array}{cc} \; & \max_{\left\{P_{g,u}\right\}}SE\\ \; & \;s.t.C1:P_{g,u}\leq P_{g,u}^{\max},\\ \; & \;\;\;C2:D_{g}\left(g,u\right)P_{g,u}-\left(2^{R_{\min}}-1\right)\\ \; & \;\;\;\;\left(\sum_{U_{q,v}\in \Omega_{g,u}}d_{g}\left(q,v\right)P_{q,v}+\sigma^{2}\right)\geq 0,\\ \; & \;\;\;C3:d_{g}\left(g,u\right)P_{g,u}-\sum_{r=u+1}^{\left|S_{g}\right|}d_{g}\left(g,r\right)P_{g,r}\geq P_{tol}, \end{array}$$

although the constraints in the original problem are simplified, the objective function in Equation (25) is in a fractional form which is still not solved. By observing the fractional form of the objective function, we can see that Equation (25) is the problem of the ratio sum, and we can consider the QT method to solve the problem of the non-convex objective function according to Lemma 2 of  [24]. The objective function $SE=\sum_{g=1}^{G}\sum_{u=1}^{\left|S_{g}\right|}\log_{2}\left(1+SINR_{g,u}\right)$ in (25) can be transformed into $QT_{\mathrm{SE}}\left(P,m\right)$ as follows

(26) $$QT_{\mathrm{SE}}\left(P,m\right)=\sum_{g=1}^{G}\sum_{u=1}^{\left|S_{g}\right|}\log\left(1+2m_{g,u}\sqrt{d_{g}\left(g,u\right)P_{g,u}}-m_{g,u}^{2}\left(\sum_{U_{q,v}\in \Omega_{g,u}}d_{g}\left(q,v\right)P_{q,v}+\sigma^{2}\right)\right),$$

where $QT_{\mathrm{SE}}\left(P,m\right)$ is the new objective function and *m* is the total set of auxiliary variables. Then, we can convert the more difficult Equation (25) into a solvable one such as (27) as follows

(27) $$\begin{array}{cc} \; & \max_{\left\{P,m\right\}}QT_{\mathrm{SE}}\left(P,m\right)\\ \; & \;s.t.C1:P_{g,u}\leq P_{g,u}^{\max},\\ \; & \;\;\;C2:D_{g}\left(g,u\right)P_{g,u}-\left(2^{R_{\min}}-1\right)\\ \; & \;\;\;\;\left(\sum_{U_{q,v}\in \Omega_{g,u}}d_{g}\left(q,v\right)P_{q,v}+\sigma^{2}\right)\geq 0,\\ \; & \;\;\;C3:d_{g}\left(g,u\right)P_{g,u}-\sum_{r=u+1}^{\left|S_{g}\right|}d_{g}\left(g,r\right)P_{g,r}\geq P_{tol}, \end{array}$$

for the problem in (27), this paper adopted an iterative optimization of the power variable *P* and the auxiliary variable *m*. When the power variable *P* is fixed, the optimal solution of $m_{g,u}$ is updated in closed form as

(28) $$m_{g,u}^{🟉}=\frac{\sqrt{d_{g}\left(g,u\right)P_{g,u}}}{\sum_{U_{q,v}\in \Omega }g_{g,u}d_{g}\left(q,v\right)P_{q,v}+\sigma^{2}},$$

when $m_{g,u}$ is fixed, (27) is converted to a problem about maximizing the power variable $QT_{\mathrm{SE}}$. Since the expression $1+2m_{g,u}\sqrt{d_{g}\left(g,u\right)P_{g,u}}-m_{g,u}^{2}\left(\sum_{U_{q,v}\in \Omega_{g,u}}d_{g}\left(q,v\right)P_{q,v}+\sigma^{2}\right)$ in $QT_{\mathrm{SE}}$ is concave and log() function is non-decreasingly concave, the problem of maximizing $QT_{\mathrm{SE}}$ with the power variable *P* is a solvable convex problem. Next, variables *P* and *m* are optimized iteratively, and the objective function $QT_{\mathrm{SE}}\left(P,m\right)$ is optimally solved when both *P* and *m* reach their optimal values. The specific power allocation algorithm flow is provided in Algorithm 3. The algorithm is essentially a block coordinate ascent algorithm that converges to a fixed point due to the concave–convex form. Details of the convergence proof are provided in  [24]. (Convergence condition).
**Algorithm 3** The Quadratic Transform Power Allocation Scheme for SE**Input:** The user grouping scheme: $U=\left\{U_{1},U_{2},\ldots ,U_{G}\right\}$, $F_{RF}$, $F_{BB}$, $h_{g,u}$.**Output:** Power Allocation $P_{g,u}$.  repeat  Update $m_{g,u}^{🟉}$ by (28);  Update $P_{g,u}$ by solving the convex optimization problem (27) for fixed *m*;  Until $QT_{\mathrm{SE}}$ converges;

### 3.4. Solution Summary and Algorithm Complexity Analysis

**Figure 3:**
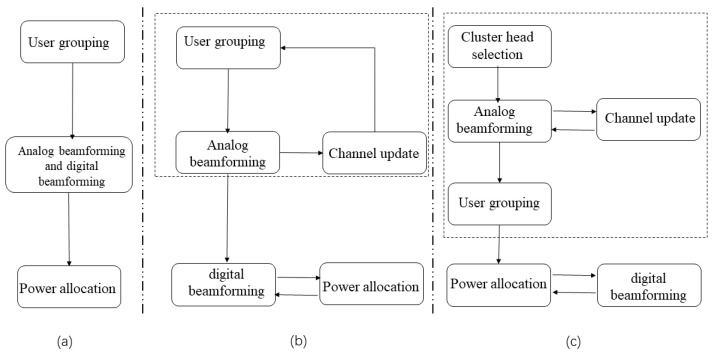
Comparison of the hybrid beamforming NOMA design procedure: (**a**) the traditional hybrid beamforming NOMA design, (**b**) the S-AGNES hybrid beamforming NOMA design (Reprinted with permission from Ref. [21]. 2021, Zhu, J), (**c**) the proposed hybrid beamforming NOMA design.

After obtaining the power allocation scheme with Algorithm 3, we designed an iterative digital beamforming and power allocation to achieve the purpose of enhancing SE. During the iteration, the power allocation may change the user order because the equivalent channel gain changes. If the strongest user is different from the previous iteration, the digital beamforming design will change significantly. Considering this, the maximum number of iterations is set in this paper, and the overall algorithmic flow framework of the uplink hybrid mmWave MIMO-NOMA system is shown in Algorithm 4. Specifically, Algorithms 1 and 2 are used for cluster head selection and user grouping to reduce the interference among users, respectively, followed by an iterative digital beamforming scheme and power allocation scheme (Algorithm 3) to maximize the SE of users, and end the iterative process when the SE converges or the number of iterations exceeds the maximum number of iterations to obtain the optimal digital precoding matrix and power allocation matrix.
**Algorithm 4** Solution Summary**Input:** The number of users *K*, channel veactors: $h_{k}$,the number of beams *G*, the maximum number of iterations: *T*. **Output:** The user grouping scheme:$U=\left\{U_{1},U_{2},\ldots ,U_{G}\right\}$, $F_{RF}$, $F_{BB}$, Power Allocation $P_{g,u}$. Perform cluster head user selection by Algorithm 1; Perform a phase-aligned analog beamforming by (16) and (17); Attributing users to clusters by Algorithm 2; repeat Calculate the digtal beamformer $F_{BB}$ by (20) and (21); Allocate power for maximizing SE by Algorithm 3; ITE←ITE+1; Until SE converges or ITE>T;


The research process of this paper is shown in Figure 3. Conventional uplink hybrid beamforming NOMA design adopted a structure of user grouping, hybrid beamforming and power allocation separation, considering each step singularly as shown in Figure 3a. However, this generated a serious beam overlap problem, which led to the degradation of SE. To better suppress the interference and address the overlapping beam problem, ref. [21] proposed the S-AGNES algorithm illustrated in Figure 3b. Ref. [21] proposed that the design of the user grouping and the analog beamforming reduces the inter-group interference when choosing a less correlated beam under a Gram–Schmidt based procedure. In contrast to the above traditional hybrid beamforming NOMA design, the S-AGNES algorithm enjoys more resource allocation flexibility and low complexity, which further helps enhance the system performance. In this paper, we considered the influence of cluster head users on user grouping and analog beamforming design while retaining the advantages of the S-AGNES algorithm in Figure 3c. Further, we designed the cluster head selection algorithm based on cluster head users and the phase-aligned analog beamforming design to improve SE by solving the inter-user interference problem.

Next, we analyzed the complexity of our proposed cluster head selection algorithm (Algorithm 1) and user grouping algorithm (Algorithm 2). For the adaptive cluster head selection algorithm, the maximum complexity was (2+2(K−1))(K−1) from Step 7 to Step 13, while the maximum complexity was 2(K−1) from Step14 to Step16. Therefore, the total algorithm complexity of Algorithm 1 was $O\left(GK^{2}\right)$. For the user grouping algorithm, Algorithm 2 is a nested loop, and the algorithm complexity can be calculated as $O\left(N_{RF}N_{BS}K\right)$.

## 4. Simulation Results

### 4.1. Performance Metrics

In this section, we demonstrate the effectiveness of the proposed algorithm by analyzing the simulation results with different parameters and compare the analysis with existing algorithms. We consider a multi-user single-antenna scenario, where the BS is equipped with the number of $N_{BS}=64$, the resolution of the phase shifter is 4 bits, and K users are divided into $G=N_{RF}$. For user $U_{g,u}$ channel path and gain settings, we set the number of paths L=6. One LoS channel and five NLoS channels were included in all path numbers, and their channel gains were set to $\alpha_{g,j,l}∼CN\left(0,1\right)$, l=1,…,L. The channel is constructed by using a ULA, where $\theta_{g,u,l}\in \left[-\frac{\pi }{2},\frac{\pi }{2}\right]$, l=1,…,L. The maximum number of iterations is T=20. Simulation results are obtained by 3000 Monte Carlo simulations, and the simulation software is matlab.R2018.a. (see Table 1 for specific parameters).

### 4.2. Comparison Algorithm Analysis

The algorithms used for simulation comparison in this paper are as follows (Pro is the scheme proposed in this paper)

- Pro: Algorithms 1 and 2 for user grouping, and ZF digital beamforming and Algorithm 3 for digital beamforming and power allocation to maximize system SE.
- SUC: Hierarchical clustering algorithm and S-AGNES algorithm for user grouping and analog beamforming design [21], and ZF precoding method and Algorithm 3 for digital beamforming and power allocation to maximize system SE.
- AVE: User grouping algorithm based on the difference in channel gain [9], D-AGNES algorithm for analog beamforming design [21], and ZF precoding method and Algorithm 3 for digital beamforming and power allocation to maximize system SE.
- K-means: User grouping algorithm uses the K-means algorithm [13], the analog beamforming design uses the S-AGNES algorithm, and the digital beamforming and power allocation uses the ZF precoding method and Algorithm 3 to maximize the system SE.
- OMA: The traditional orthogonal multiple access (OMA) algorithm serves only users in the same time domain and is implemented through a ZF precoding and power allocation design, but unlike other algorithms it does not take into account intra-group interference and the decoding order.

**Figure 4:**
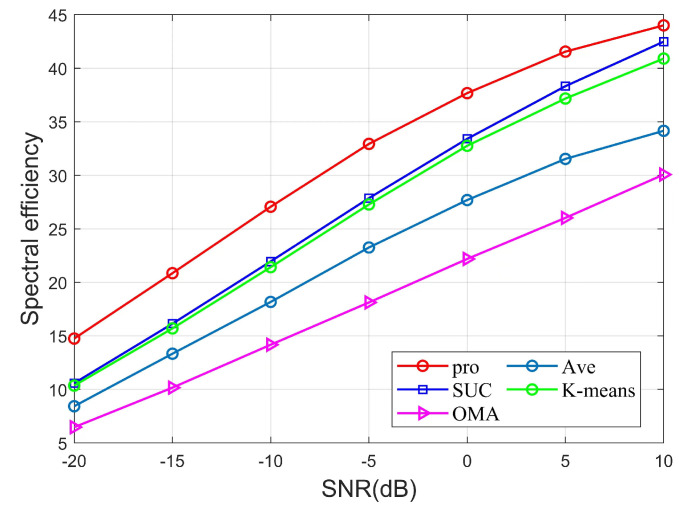
SE versus SNR of different algorithms.

Figure 4 shows the variation of signal-to-noise ratio (SNR) versus SE for different algorithms in an uplink mmWave MIMO-NOMA system with fixed parameters K=9, $N_{RF}=4$, $P_{\max}=24\;\mathrm{mW}$, $P_{\mathrm{tol}}=2\;\mathrm{mW}$, $R_{\min}$= 0.01 bps/Hz. The figure shows that the SE of different algorithms increases with the increase in SNR and tends to be flat at high SNR. Among the different algorithms, the OMA algorithm is significantly weaker than the NOMA scheme algorithm for the same SNR because OMA can only serve users in the same time-frequency domain. In the NOMA scheme, AVE considers the channel gain difference as the user grouping metric, but the SE is significantly worse than the other NOMA algorithms because it is not applicable to mmWave systems. Among the remaining algorithms, the proposed scheme in this paper performs significantly better than the SUC and K-means schemes in terms of SE at SNR [−20,10]. Because SUC uses a suboptimal hierarchical clustering user grouping algorithm, the user grouping results of the K-means scheme are affected by the initial cluster head selection, and the simulated beamforming designs of these schemes do not complete the optimal phase alignment, and only the locally optimal simulated beamforming designs are obtained. Therefore, we can see that the proposed scheme in this paper has better SE performance with the same SNR.

### 4.3. Simulation Analysis

**Figure 5:**
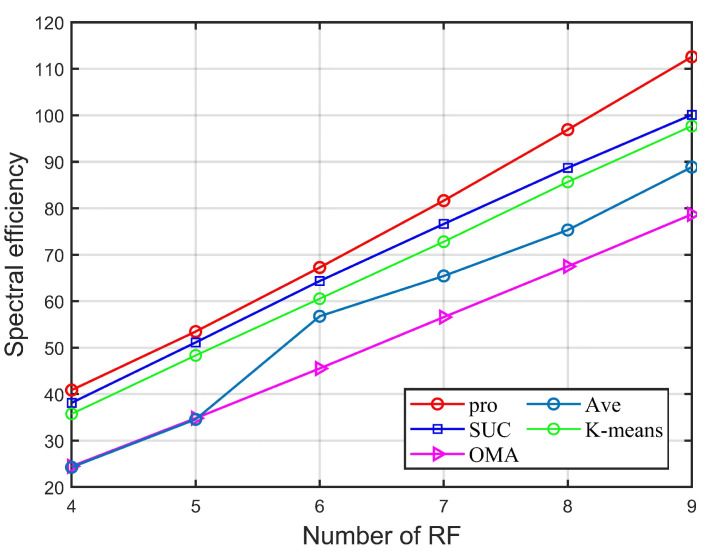
SE versus the number of the RF chains of different algorithms.

Figure 5 shows the variation of the RF chain number and SE for different algorithms with fixed parameters K=12, SNR=10dB, $P_{\max}=24\;\mathrm{mW}$, $P_{\mathrm{tol}}=1\;\mathrm{mW}$, $R_{\min}=0.01\mathrm{bps}/\mathrm{Hz}$. With the increasing number of RF chains, the SE performance of all algorithms, except AVE algorithm, increases linearly, and the SE performance of AVE algorithm increases abruptly after the number of RF equals six, because the number of RF chains is greater than six and the number of user groups per group will be less than two, which is less affected by the user grouping scheme. The proposed scheme in this paper has stable SE superiority regardless of the change in the number of RF. When the number of RF chains is five, the algorithm of this paper outperforms the SUC algorithm, the K-means algorithm, the Ave algorithm, and OMA by 4.6%, 10.6%, 53.5%, and 54.6%, respectively.

**Figure 6:**
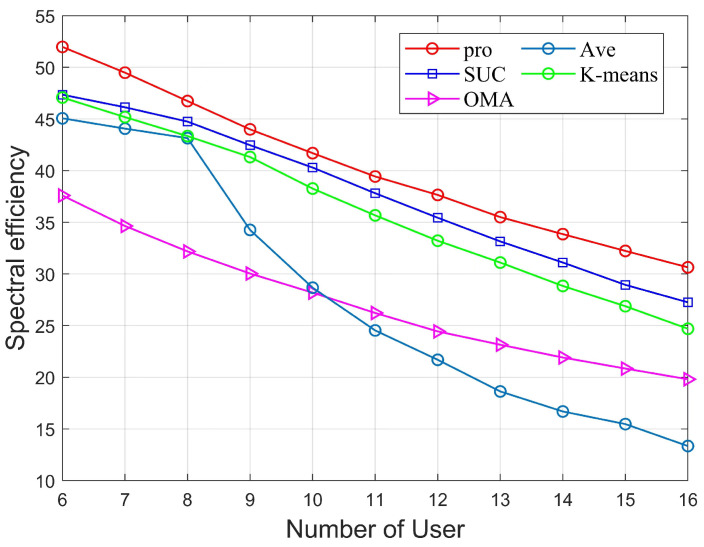
SE versus $P_{\mathrm{tol}}$ of different algorithms.

Figure 6 shows the variation of K versus SE for different algorithms in the uplink mmWave MIMO-NOMA system with fixed parameters $N_{RF}=4$, SNR=10dB, $P_{\max}=24\;\mathrm{mW}$, $P_{\mathrm{tol}}=2\;\mathrm{mW}$, $R_{\min}$ = 0.01 bps/Hz. Apparently, as the number of users increases, the inter-user interference increases and the allocated power for each user decreases. Therefore, the SE shows a decreasing trend. The OMA system performs the worst compared to the NOMA system because the OMA system can only serve users in the same time channel. The AVE algorithm, on the other hand, proves to be ineffective as the SE performance decreases rapidly when the number of users is twice the number of groups G. Among the remaining NOMA schemes, the proposed scheme clearly outperforms SUC and K-means, which proves that this scheme can guarantee reasonable user grouping and perform optimally in terms of SE.

**Figure 7:**
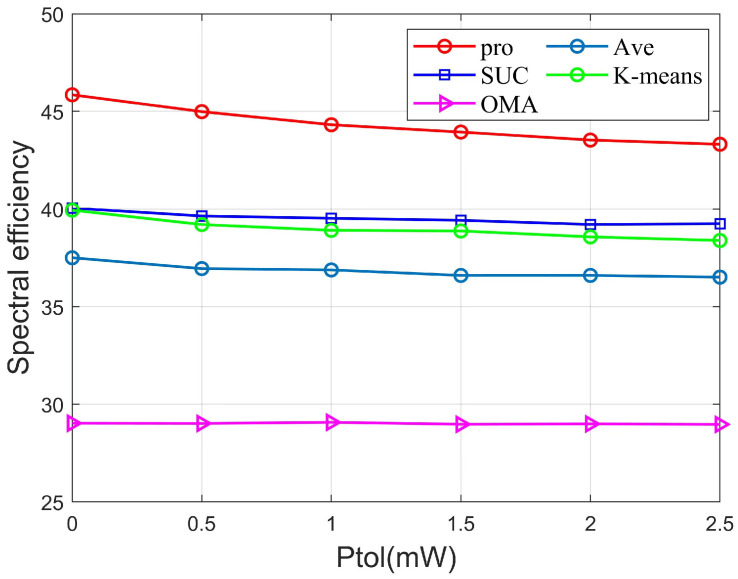
SE versus $P_{\mathrm{tol}}$ of different algorithms.

Figure 7 shows the variation of $P_{\mathrm{tol}}$ and SE for different algorithms with fixed parameters K=7, SNR=3dB, $P_{\max}=20\;\mathrm{mW}$, $R_{\min}=$ 0.01 bps/Hz. From the above figure, it can be seen that the NOMA scheme decreases as the power interval increases, while the OMA scheme remains constant as the power interval increases. When $P_{\mathrm{tol}}$ is 1, the algorithm of this paper outperforms the SUC algorithm, the K-means algorithm, the Ave algorithm, and OMA by 12.1%, 13.9%, 20.2%, and 52.5%, respectively. When $P_{\mathrm{tol}}$ is 2.5, the algorithm of this paper outperforms the SUC algorithm, the K-means algorithm, the Ave algorithm, and OMA by 10.4%, 10.2%, 18.7%, and 49.5%, respectively. We can see that the proposed scheme performs best compared to other schemes.

**Figure 8:**
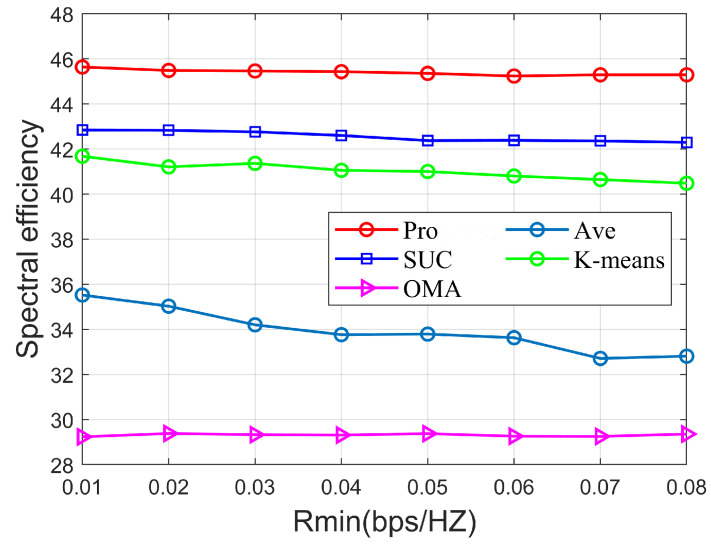
SE versus $R_{\min}$ of different algorithms.

Figure 8 shows the variation of and SE for different algorithms with fixed parameters K=9, $N_{RF}=4$, SNR=5dB, $P_{\max}=40\;\mathrm{mW}$, $P_{\mathrm{tol}}=1\;\mathrm{mW}$. From the above figure, it can be seen that the NOMA scheme decreases as the minimum rate requirement increases. This is because when the user minimum rate requirement increases, the system needs to allocate more power to weak users, resulting in a reduction in the SE of the system. From the figure, we can see that the algorithm in this paper always outperforms the SUC algorithm, the K-means algorithm, the Ave algorithm, and OMA, regardless of how the user minimum rate constraint changes.

## 5. Conclusions and Future Work

### 5.1. Conclusions

In this paper, we addressed the problem of maximizing the SE of an uplink mmWave MIMO-NOMA system. To maximize the SE of system, cluster head selection, user grouping, hybrid beamforming, and power allocation were carefully designed. Firstly, we introduced the cluster head concept to propose an adaptive cluster head selection algorithm. Then, a channel-aligned analog beamforming scheme was designed based on the selected cluster heads. A user grouping algorithm was designed to suppress the inter-user interference problem based on the user-equivalent channel correlation. Subsequently, the QT method was used to transform the power allocation problem from a nonconvex problem to a convex one by considering all relevant constraints. Finally, the optimal user power allocation and digital beamforming design were obtained by optimizing the power and digital beamforming. The simulation results show that the proposed scheme had a higher SE than SUC, K-means, Ave, and conventional OMA schemes.

### 5.2. Future Work

The proposed scheme in this paper is suitable for the uplink hybrid mmWave MIMO-NOMA system and has great advantages in improving the SE compared to the existing methods. In terms of the system, this paper used a multi-user single-antenna system, which can be extended to a multi-user multi-antenna system scenario in the future. In the scheme design, the selection of cluster head users greatly impacts overall performance of the system. The future direction of work will consider using a cluster headless scheme design or a better cluster head selection scheme. 

## Figures and Tables

**Table 1:** Simulation Setup and Parameters.

Parameter	Value
Number of scatterers *L*	6
Number of anntennas $N_{BS}$	64
The resolution of phase shifter *B*	4
Number of maximum iteration T	20
The range of SNR(dB)	[−20, 10]
The range of number of user	[6, 16]
The range of number of RF chains	[4, 9]

## Data Availability

Not applicable.
